# Social regulation of gene expression in human leukocytes

**DOI:** 10.1186/gb-2007-8-9-r189

**Published:** 2007-09-13

**Authors:** Steve W Cole, Louise C Hawkley, Jesusa M Arevalo, Caroline Y Sung, Robert M Rose, John T Cacioppo

**Affiliations:** 1 Department of Medicine, Division of Hematology-Oncology, UCLA School of Medicine, Los Angeles CA 90095-1678, USA; 2 UCLA AIDS Institute, UCLA Molecular Biology Institute, Jonsson Comprehensive Cancer Center; 3 Norman Cousins Center; 4 Department of Psychology, and Center for Cognitive and Social Neuroscience, University of Chicago; 5 Institute for Medical Humanities, University of Texas Medical Branch at Galveston, and the John D and Catherine T MacArthur Foundation

## Abstract

**Background:**

Social environmental influences on human health are well established in the epidemiology literature, but their functional genomic mechanisms are unclear. The present study analyzed genome-wide transcriptional activity in people who chronically experienced high versus low levels of subjective social isolation (loneliness) to assess alterations in the activity of transcription control pathways that might contribute to increased adverse health outcomes in social isolates.

**Results:**

DNA microarray analysis identified 209 genes that were differentially expressed in circulating leukocytes from 14 high- versus low-lonely individuals, including up-regulation of genes involved in immune activation, transcription control, and cell proliferation, and down-regulation of genes supporting mature B lymphocyte function and type I interferon response. Promoter-based bioinformatic analyses showed under-expression of genes bearing anti-inflammatory glucocorticoid response elements (GREs; *p *= 0.032) and over-expression of genes bearing response elements for pro-inflammatory NF-κB/Rel transcription factors (*p *= 0.011). This reciprocal shift in pro- and anti-inflammatory signaling was not attributable to differences in circulating cortisol levels, or to other demographic, psychological, or medical characteristics. Additional transcription control pathways showing differential activity in bioinformatic analyses included the CREB/ATF, JAK/STAT, IRF1, C/EBP, Oct, and GATA pathways.

**Conclusion:**

These data provide the first indication that human genome-wide transcriptional activity is altered in association with a social epidemiological risk factor. Impaired transcription of glucocorticoid response genes and increased activity of pro-inflammatory transcription control pathways provide a functional genomic explanation for elevated risk of inflammatory disease in individuals who experience chronically high levels of subjective social isolation.

## Abstract 2

Analysis of differentially expressed in circulating leukocytes from people who chronically experienced high versus low levels of subjective social isolation (loneliness) revealed over-expression of some anti-inflammatory genes and under-expression of some pro-inflammatory genes.

## Background

A large body of epidemiological research has linked characteristics of the social environment to human physical health [1,2], but the genomic mechanisms of these effects remain largely unexplored. One of the most robust social risk factors involves the number and quality of an individual's close personal relationships. People who are socially isolated have increased risk of all-cause mortality [1,2], and several specific infectious, neoplastic, and cardiovascular diseases [3-6]. The biological basis for these epidemiological findings is poorly understood, in part because it is unclear whether the effects of social isolation stem predominantly from the objective deprivation of instrumental social support (for example, physical, cognitive, or economic assistance), or from the biological consequences of the experienced threat and dysphoria associated with subjective social isolation (that is, loneliness). Few epidemiological studies have clearly distinguished between objective and subjective social isolation, but among those that have, some evidence supports a significant contribution from each aspect [1,5,7-10]. However, the physiological signaling pathways by which these dynamics impact the pathobiology of disease remain poorly understood.

Experimental manipulation of social contact in animals can activate neuroendocrine signaling pathways [11-14], which have the potential to regulate gene expression in both pathogens (viruses, bacteria, tumors) and host immune responses [4,14-26]. No experimental studies have analyzed the transcriptional impact of chronic social isolation in humans, but data from observational studies suggest that subjective social isolation (loneliness) is associated with increased circulating levels of the stress hormone cortisol [27-30]. This adrenal glucocorticoid can regulate a wide variety of physiological processes via nuclear hormone receptor-mediated control of gene transcription [31]. Cortisol activation of the glucocorticoid receptor (GR) exerts broad anti-inflammatory effects by inhibiting nuclear factor (NF)-κB/Rel transcription factors and other pro-inflammatory signaling pathways (for example, the Janus kinase-signal transducer and activator of transcription (JAK/STAT) and interferon response factor (IRF) signaling) [32,33]. However, increased cortisol levels in chronically lonely individuals is paradoxical in light of the fact that most isolation-linked diseases are driven by increased inflammation (for example, lentiviral replication, atherosclerosis, and solid tissue malignancies) [34-36]. Given the broad anti-inflammatory effects of glucocorticoids, chronically lonely people with elevated cortisol levels should be relatively protected from inflammation-mediated disease rather than having the increased risk empirically observed.

One possible explanation for inflammation-related disease in individuals with high cortisol levels involves functional desensitization of the GR pathway that mediates transcriptional response to glucocorticoids. Several molecular mechanisms have been shown to render cells insensitive to the anti-inflammatory effects of glucocorticoids *in vitro*, including decreased expression of the GR *NR3C1 *gene, post-translational modification of GR protein, increased expression of GR antagonists, and decreased activity of GR transcription cofactors [37]. In both human and animal models, prolonged stress has been linked to reduced cellular expression of *NR3C1 *and increased cellular resistance to glucocorticoid inhibition of pro-inflammatory cytokine responses [37-40]. It is conceivable, therefore, that pro-inflammatory signaling persists in socially isolated people with high cortisol levels because impaired GR-mediated signal transduction prevents the cellular genome from effectively 'hearing' the anti-inflammatory signal sent by circulating glucocorticoids.

The present study utilizes an *in vivo *genomics-based strategy to identify genes that are differentially expressed in the immune system of people who experience chronically high levels of subjective isolation (loneliness), and to define the upstream transcription-control pathways that mediate those differences. Bioinformatic analyses of differentially expressing promoters [41,42] test the specific hypotheses that immune cells from high-lonely individuals show *in vivo*, under basal physiological conditions: 1.) decreased activity of the anti-inflammatory glucocorticoid transcription control pathway; and 2.) increased activity of the pro-inflammatory NF-κB/Rel pathway. Results reveal a distinct 'transcriptional fingerprint' of experienced social isolation that includes genomic indications of immune activation, and a reciprocal shift in the activity of pro- and anti-inflammatory transcription control pathways that shape global gene expression in the human immune system.

## Results

### Sample characteristics

Global gene transcription profiles were assessed in circulating leukocytes from 14 individuals participating in the population-based Chicago Health Aging and Social Relations Study (CHASRS) [43]. Individuals experiencing high levels of subjective social isolation were identified by scores in the top 15% of the UCLA Loneliness Scale, and an age-, gender-, and race-matched comparison group of individuals experiencing subjective social integration was defined by Loneliness scores in the bottom 15% [28]. Table 1 provides demographic characteristics of each group, along with medical, behavioral, and psychosocial characteristics. This sample was composed of older American adults (median age 55 years), with diverse ethnic backgrounds (50% Anglo-American, 43% African-American, and 7% Hispanic/Latino), and varying socioeconomic status (median household income of $62,500 per year, range $25,000 to $150,000). A majority of study participants were female (78% female, 22% male), and measured levels of loneliness were stable over the 3 years prior to this gene expression analysis (1-year test-retest correlations averaged *r *= 0.90; intraclass correlation over all 3 years = 0.937, *p *< 0.0001). Sample selection procedures generated the expected difference in average levels of experienced social isolation (difference in UCLA Loneliness scores, *p *= 0.0002; Table 1). High-lonely participants did not differ from low-lonely controls on any of the medical or behavioral dimensions analyzed, but they were slightly younger (4 year difference, *p *= 0.011), had a lower household income (median $45,000 per year versus $87,500, *p *= 0.010), and reported higher levels of perceived stress (*p *= 0.023) and depressive symptoms (50% ≥ CEpidemiologic Studies Depression scale (CESD) cut-point of 10 versus 0% of controls, *p *= 0.038). These demographic and psychological differences were treated as potential confounders in subsequent analyses.

**Table 1:** Demographic, medical, behavioral, and psychosocial characteristics of study participants

Characteristic	Integrated	Isolated
**Demographic**		
Gender (% female)	75.0%	83.3%
Age (mean ± SD years)	57.5 ± 3.3	53.5 ± 1.5
Ethnicity		
Anglo-American	62.5 %	33.3 %
African-American	25.0 %	66.7 %
Hispanic	12.5 %	0.0 %
Household income (mean ± SD × $10,000 yearly)	91.6 ± 39.2	42.5 ± 17.2
Marital status (% married)	63.5 %	50.0 %
**Medical**		
Body mass index (mean ± SD)	31.8 ± 6.3	34.2 ± 7.5
Coronary artery disease (% diagnosed)	12.5 %	16.7 %
High blood pressure (% diagnosed)	50.0 %	66.7 %
Diabetes (% diagnosed)	12.5 %	33.3 %
Kidney disease (% diagnosed)	12.5 %	33.3 %
Liver disease (% diagnosed)	0.0 %	0.0 %
Respiratory disease (% diagnosed)	12.5 %	0.0 %
Anti-inflammatory medications (% prescribed)	25.0 %	33.3 %
Statin medications (% prescribed)	25.0 %	33.3 %
Anti-diabetic medications (% prescribed)	12.5 %	33.3 %
Beta-blocker medications (% prescribed)	12.5 %	33.3 %
Psychotropic medications (% prescribed)	12.5 %	16.7 %
**Behavioral**		
Regular exercise (% reporting)	100.0 %	100.0 %
Alcohol consumption (mean drinks/week ± SD)	2.6 ± 4.1	1.8 ± 3.3
Smoking (% smokers)	0.0 %	0.0 %
**Psychosocial**		
UCLA Loneliness (mean ± SD)	29.9 ± 5.1	46.0 ± 5.6
Depressive symptoms (CESD mean ± SD)	1.9 ± 2.8	15.3 ± 11.9
Stress (Perceived Stress Scale mean ± SD)	7.5 ± 6.6	15.8 ± 5.3
Hostility (Cook-Medley Hostility Inventory mean ± SD)	11.1 ± 7.0	17.2 ± 8.2

SD, standard deviation.

### Differential gene expression

**Figure 1:**
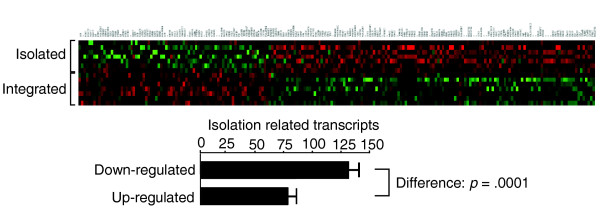
Differential gene expression in high- versus low-lonely individuals. Genome-wide transcriptional profiles were assessed in peripheral blood leukocyte RNA samples collected from individuals in the top and bottom 15% of the distribution of subjective social isolation. Analysis by Affymetrix U133A high-density oligonucleotide arrays identified 209 transcripts showing >30% difference in mean expression levels across groups (green = over-expression in high-lonely, red = under-expression). High subjective social isolation is associated with a statistically significant net reduction in the number of expressed genes (131 down-regulated versus 78 up-regulated, *p *value by exact binomial test).

To assess leukocyte transcriptional alterations associated with chronic loneliness, we carried out global gene expression profiling using Affymetrix U133A high-density oligonucleotide arrays to survey 22,283 mRNA transcripts. Peripheral blood mononuclear cells were collected by antecubital venipuncture during the fourth or fifth yearly CHASRS visit. Figure 1 presents the 'transcriptional fingerprint' of subjective social isolation in human luekocytes, with green intensity indicating the magnitude of a gene's relative over-expression in high-lonely indivdiuals, and red intensity denoting the magnitude of relative under-expression. A total of 209 transcripts were differentially expressed, representing 144 distinct named human genes (listed in Additional data file 1). Of these, 78 (37%) were over-expressed in high-lonely individuals, and 131 (63%) were under-expressed, suggesting a net repressive effect on the leukocyte transcriptome (difference *p *< 0.0001 by binomial test).

Genes showing over-expression in leukocytes from high-lonely individuals included multiple transcription factors controlling cell growth and differentiation (*EGR1*, *EGR3*, *FOSB*, *BHLHB2*,*EP400*), as well as regulators of chromatin structure (the DNA topoisomerase *TOP2B*, the *ARID1A *and *SMARCC1 *components of the SWI/SNF chromatin remodeling complex, histone *H2AFV*, and the histone acetyltransferase *MYST3*). These findings suggest that social isolation might potentially influence basic gene-regulatory processes involved in cell growth and differentiation. Consistent with that hypothesis, over-expressed genes also included molecules promoting cell cycle progression (*CDC25B*, *G0S2*, *MYBL1*, *DVL3*, *BTG2*, *ARFGEF2*), enzymes involved in nucleotide and protein biosynthesis (*PPAT*, *MTRR*), cytoskeletal remodeling factors (*DCTN1*, *KIF21B*, *RPH3A*), and factors involved in processing and nuclear export of RNA (*NXF3*, *HNRPL*, *DDX17*, *SFPQ*). In the context of leukocytes, cell cycling plays a key role in immune activation and proliferation. Several other indications of immune activation emerged in the set of over-expressed genes, including pro-inflammatory cytokines, chemokines, granzymes, proteinases, and receptors for inflammatory mediators (*IL1B*, *IL8*, *IL8RB*, *IL10RA*, *PTGDR*,*KLRC3*,*NKTR*,*GZMK*, and multiple *HLADR *genes), as well as the master regulator of prostagladin synthesis, cyclo-oxygenase 2 (*COX2*/*PTGS2*). Additional indicators of immune cell activation that were over-expressed included the immediate-early response gene *IER2*, components of the insulin-like growth factor signaling pathway (*IGF2R*, *IGFBP3*), and activation-induced counter-regulators involved in pathway desensitization (*TNFAIP*, *DUSP1*, *RGS1*) and receptor shedding (*MAN2C1*, *ADAM8*, *ARTS-1*). GOstat bioinformatic analysis [44] confirmed the functional theme of a highly activated, proliferative phenotype in circulating leukocytes from socially isolated individuals by identifying over-representation of Gene Ontology (GO) annotations involving immune response and inflammation (GO:0009607, GO:0006952, GO:0006955; GO:0009613), stress response (GO: 0006950), antigen processing (GO:0019886, GO:0019884, GO:0045012), chemokine and cytokine activity (GO:0042379, GO:0008009, GO:0005125; GO:0001965), deoxyribonucleotide metabolism (GO:0009262), and nucleosome activity (GO:0000786) (all *p *< 0.05).

Analysis of under-expressed genes complemented the portrait of generalized immune activation in showing reduced expression of cell cycle inhibitors (*CDKN1C*, *CSPG6*, *p15*/*PAF*/*KIAA0101*, *SPARC*, *FKBP5*), apoptosis-related genes (*MCL1*, *BIRC1*, *HSXIAPAF1*, and *TNFSF10*/TRAIL), the antiproliferative cytokine TGF-β (*TGFB1*), and the NF-κB inhibitor Apo J (*CLU*). However, several specific dimensions of immune response showed selective repression against this generalized backdrop of immune activation. Genes involved in type I interferon response were down-regulated, including the signal transducer *STAT1 *and the interferon response genes 2',5'-oligoadenylate synthetase 1 (*OAS1*), interferon-stimulated gene 12 (*IFI27*), interferon-induced protein 6-16 (*G1P3*), and interferon-inducible proteins 15 and 44 (*G1P2*, *IFI44*). Also down-regulated were genes encoding immunoglobulin light, joining, and heavy region chains (*IGLC2*, *IGLL1*, *IGK*/*IGKC*/*IGKGV*, *IGJ*, *IGLJ3*, *IGHG1*, *IGHM*), B lymphocyte maturation markers (*CD79B*, *CD269*/*TNFRSF17*), and transcription fators involved in B cell differentiation (*Ikaros*/*ZNFN1A1*, *POU2AF1*). Thus, the circulating leukocyte complement in chronically lonely individuals shows broad genomic indications of immune activation and pro-inflammatory signaling accompanied by selective reductions in mature B lymphocyte function and innate antiviral responses.

Eight transcripts characteristic of the two key functional GO categories showing differential activity (immune activation and transcriptional control) were selected for independent verification of differential expression by real-time RT-PCR. Results were concordant with microarray indications in seven instances, with RT-PCR confirming over-expression of *IL1B*,*IL8*,*PTGS2*/*COX2*, *FOSB*, *EGR1*, *CDC25B*, and under-expression of *G1P3 *(MANOVA difference in all transcripts simultaneously, multivariate *p *< 0.0001, and *p *< 0.05 for each transcript analyzed individually; Additional data file 2). Both micorarray data and quantitative RT-PCR found the GR *NR3C1 *gene to be transcribed at comparable levels in leukocytes from high- versus low-lonely individuals (difference <4% by RT-PCR, *p *= 0.815, and <15% by microarray, *p *= 0.112). Expression of CD3 (*CD3G*, *CD3D*, *CD3E*, *CD3Z*), CD8 (*CD8A*, *CD8B1*), *CD4*, CD56 (*NCAM1*), *CD19*, and *CD14 *were also comparable (all differences <10% in magnitude, all *p *> 0.20). Thus, observed differences in mononuclear cell gene expression do not stem from variations in the relative prevalence of leukocyte subset mRNAs within the circulating mononuclear cell RNA pool [45-47].

#### C-reactive protein

To verify the portrait of increased inflammatory signaling that emerged from functional genomic analyses, we assayed circulating levels of C-reactive protein (CRP) in blood samples obtained during study years 2, 3, and 4. In mixed effect linear model analyses (Year × Loneliness factorial design), high-lonely individuals showed average CRP values approximately twice those of low-lonely participants (mean = 1.33 ± 0.22 mg/l versus 0.65 ± 0.22; Loneliness main effect, *p *= 0.0014).

### Transcription control pathways

#### Cortisol levels

To determine whether the increased transcription of immune activation genes in high-lonely individuals might stem from reduced circulating levels of the anti-inflammatory glucocorticoid cortisol, we assayed salivary cortisol concentrations immediately upon waking, 30 minutes later, and at bedtime on three consecutive days during study years 1, 3 and 4. Mixed effect linear model analysis (Time × Loneliness factorial design) indicated no significant reduction in average daily salivary cortisol levels in high-lonely individuals (mean = 4.061 ± 0.417 μg/dl versus 4.541 ± 0.342 for low-lonely; Loneliness main effect, *p *= 0.2374). However, high-lonely individuals did show less diurnal decline in circulating cortisol levels than did low-lonely participants (Time × Isolation interaction, *p *= 0.0474; means in Table 2). This difference was driven by slightly higher evening levels of cortisol; change in salivary cortisol concentration from 0 to 0.5 h post-awakening did not differ significantly across groups (*p *= 0.8610), but 0-16 h and 0.5-16 h declines were both less pronounced in high-lonely individuals (*p *= 0.0260 and 0.0396, respectively). Blunted diurnal cortisol rhythm was also observed in analyses treating measurement occasion as a linear effect of hours post-awakening (slope = -0.202 ± 0.033 μg/dl/h for high-lonely versus -0.301 ± 0.042 for low-lonely; Hour × Loneliness interaction, *p *= 0.0119).

**Table 2:** Salivary cortisol levels in high- versus low-lonely individuals

	Hour			
		
	Wake (0 h)	30 min (0.5 h)	Bedtime (16 h)	Diurnal slope
Low lonely	5.44 ± 0.42*	6.82 ± 0.42	1.36 ± 0.42	-0.301 ± 0.042^†^
High lonely	4.42 ± 0.53	5.87 ± 0.53	1.90 ± 0.53	-0.202 ± 0.033

*Mean ± standard error μg/dl saliva; ^†^μg/dl/h.

#### Glucocorticoid transcriptional response

**Figure 2:**
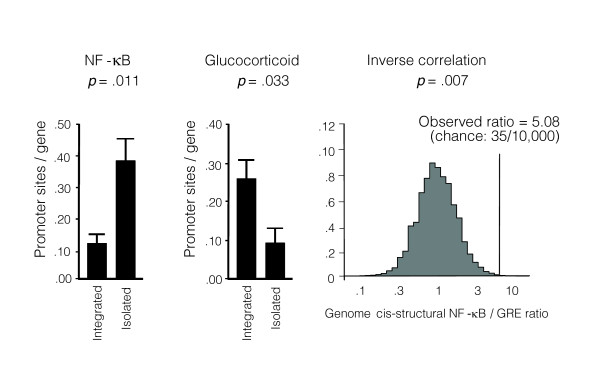
Transcriptional activity of GR and NF-κB signaling pathways. TELiS bioinformatics analysis assessed *trans*-activational activity based on the relative prevalence of GR and NF-κB response elements in the promoters of all 209 transcripts over-expressed in high- versus low-lonely individuals (data represent mean ± standard error prevalence of response elements within promoters from each group). Contributions of in-*trans *regulatory influences to the observed inverse skew of NF-κB and GR response elements within differentially expressing promoters was tested by comparison to a null distribution of genome-wide DNA *cis*-structural associations generated by 10,000 random samples of 209 transcripts assayed by Affymetrix U133A arrays.

To determine whether the increased transcription of immune activation genes in social isolates might stem from reductions in GR-mediated transcriptional activity (despite broadly similar levels of its cortisol ligand), we performed Transcription Element Listening System (TELiS) bioinformatics analysis of promoter DNA sequences [41]. Analyses of glucocorticoid response element (GRE) prevalence in differentially expressing promoters indicated significant under-expression of GR target genes in leukocytes from socially isolated individuals. The prevalence of TRANSFAC V$GR_Q6 transcription factor-binding motifs (TFBMs) was 63% lower in regulatory sequences of genes over-expressed in high-lonely individuals versus genes over-expressed in low-lonely individuals (mean = 0.257 ± 0.063 versus 0.094 ± 0.041; *p *= 0.0325). Similar results emerged in sensitivity analyses that parametrically varied the length of promoter sequence scanned (-300 bp, -600 bp, -1,000 to +200 bp from transcription start site) and the stringency of promoter sequence match to the TFBM consensus motif (MatSim score = 0.80, 0.90, 0.95), with an average 0.53-fold (± 0.10) difference across all 9 parametric combinations (*p *= 0.0020). Similar results also emerged from sensitivity analyses utilizing a more stringent definition of differential gene expression (50% difference in expression, corresponding to 5% false discovery rate (FDR)), with an average 0.82-fold (± 0.09) difference (*p *= 0.0009). These results are consistent with a significant reduction in GR-mediated transcriptional activity in leukocytes from socially isolated indivdiuals (Figure 2).

#### NF-κB transcriptional response

To determine whether increased transcription of immune activation genes in high-lonely individuals might stem from increased activity of the pro-inflammatory NF-κB transcription control pathway, TELiS bioinformatics analyses also assessed the relative distribution of NF-κB/Rel response elements in differentially expressing promoters. Results showed a 2.9-fold greater prevalence of NF-κB/Rel motifs among promoters of genes over-expressed in socially isolated individuals relative to those over-expressed in socially integrated individuals (TRANSFAC V$CREL_01 motif: average 0.129 ± standard error 0.040 sites/promoter for genes over-expressed in socially integrated individuals versus 0.377 ± 0.086 in isolated; difference *p *= 0.0108 by *t*-test) (Figure 2). Similar results emerged in parametric sensitivity analyses, with an average 2.69-fold (± 0.47) difference across all 9 parametric combinations of promoter length and motif match stringency (*p *= 0.0069), and an average 1.30-fold (± 0.09) difference in analyses utilizing 5% FDR stringency in gene selection (*p *< 0.0001).

#### GR cross-regulation of NF-κB

Bioinformatic indications of a simulatneous decrease in transcription of GR target genes (.53-fold difference) and an increase in transcription of NF-κB/Rel target genes (2.69-fold difference) is consistent with the known cross-inhibition of those two pathways [37]. Combined TELiS analysis of both pathways yielded a net 5.08-fold skew in promoter TFBM distributions (ratio = 2.69/0.53). To ensure that this skewed motif prevalence reflected the effects of reciprocal signaling in *trans*, rather than an inverse relationship between NF-κB versus GRE TFBMs in the *cis*-regulatory structure of the human gene population, we assessed the whole-genome distribution of NF-κB/GRE prevalence ratios by computing TFBM prevalence ratios in a similarly sized random sample of all assayed genes (rather than the specific subset showing differential expression in social isolates). Relative to the distribution of *cis*-structural TFBM prevalence ratios generated by 10,000 random samples of 209 transcripts drawn from the 22,283 transcripts assayed by the Affymetrix U133A microarray, the 5.08-fold NF-κB/GRE skew observed in the promoters of differentially expressed genes was substantially greater than expected by chance under the genome-wide null distribution (Figure 2; two-tailed *p *= 0.0070). Similar results emerged in analyses estimating reciprocal NF-κB/GRE skew from the high-stringency 5% FDR criterion for differential gene expression (*p *= 0.014). Thus, the inverse correlation between NF-κB/Rel and GRE TFBM prevalence in promoters of isolation-linked genes likely reflects *trans*-activational influences rather than a population-level bias in the *cis*-structure of human promoters.

#### Medical and behavioral confounders

To determine whether loneliness-related alterations in the expression of NF-κB- and GRE-responsive genes might stem from variations in the prevalence of distinct leukocyte subsets within the mononuclear cell RNA pool [45-47], analyses of covariance (ANCOVA) were employed to adjust gene expression profiles for the relative prevalence of CD3, CD4, CD8, CD14, CD19, and CD56 transcripts prior to the identification of differentially expressed genes and subsequent TFBM bioinformatics. Consistent with the findings reported above ('Differential gene expression'), results continued to show a significant increase in NF-κB/GRE prevalence ratio in promoters from genes over-expressed in leukocytes from socially isolated individuals (all *p *≤ 0.0239).

To determine whether loneliness-related increases in NF-κB/GRE ratios might stem from correlated differences in other demographic, medical, psychological, social, or behavioral characteristics, additional ANCOVAs adjusted gene expression profiles for those characteristics prior to bioinformatic analysis of transcription control pathways. NF-κB/GRE ratios remained significantly elevated in high-lonely individuals following control for demographic factors (including age, gender, race, marital status, and household income; all *p *≤ 0.0249), other established psychological risk factors for disease (including depression, perceived stress, and hostility; all *p *≤ 0.0334), medical conditions (including hypertension, coronary artery disease, myocardial infarct, emphysema, rheumatoid arthritis, cancer, ulcers, and strokes or other neurological disorders; all *p *≤ 0.0395), other biomedical parameters (including body mass index and use of statins, beta-blockers, anti-inflammatory agents, diuretics, antidepressants, and other psychiatric medications; all *p *≤ 0.0486), and behavioral risk factors (including smoking and alcohol consumption; all *p *≤ 0.0085). Socially isolated individuals showed a nonsignificant trend toward greater diagnosed diabetes and use of anti-diabetic agents, but elevated NF-κB/GRE ratios continued to approach statistical significance despite control for those factors (both *p *≤ 0.0594).

As in previous studies [2,28], subjective social isolation (as measured by the UCLA Loneliness Scale) was only modestly correlated with the objective size of an individual's social network (as measured by the Social Network Index); r = +0.277, *p *= 0.3603. In analyses controlling for variations in objective social network density, NF-κB/GRE ratios remained significantly elevated in individuals experiencing chronically high levels of subjective social isolation (*p *= 0.0258). This result is consistent with previous findings that neuroendocrine function is more strongly related to subjective social isolation than to objective social network density [2,28].

To further distinguish between the effects of chronic loneliness and transient fluctuations in subjective social isolation (that is, state loneliness), we conducted ANCOVAs controlling for residual variance in UCLA Loneliness scores assessed during the specific study visit in which gene expression was assayed. Trait loneliness was assessed by the average UCLA score across study visits 1-3 (the basis for group classification), and state loneliness was quantified as the difference betweeen an individual's UCLA score at visit 4 or 5 (gene expression visit) and trait loneliness. Despite control for variations in state loneliness, gene expression profiles contrasting high versus low trait loneliness continued to show a significant elevation in the expression of NF-κB-/GRE-responsive genes (*p *= 0.0120).

#### Additional signaling pathways

**Figure 3:**
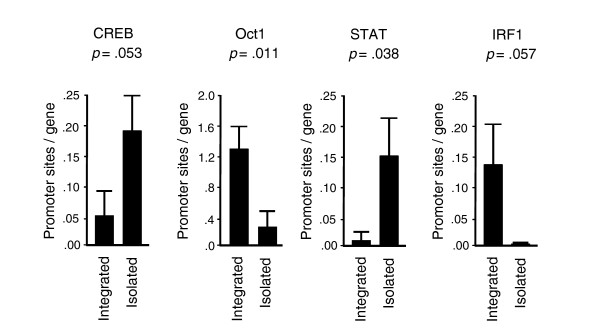
Transcription control pathways differentially active in high- versus low-lonely individuals. Data represent the mean (± standard error) prevalence of transcription factor-binding motifs in primary TELiS bioinformatics analysis of genes over-expressed in leukocytes from high- versus low-lonely individuals.

To identify additional transcription control pathways that might contribute to isolation-linked differences in genomic activity, we carried out exploratory TELiS analyses of 190 other vertebrate TFBM motifs from the TRANSFAC database (Table 3). Two findings that emerged consistently across parameteric variations in analysis parameters involved increased activity of the CREB/ATF family of transcription factors (average 2.2-fold increase in promoter TFBM prevalence, *p *= 0.0044) and decreased activity of Octamer (Oct) family transcription factors (average 62.2% reduction, *p *= 0.0004) (Figure 3). Increased CREB/ATF activity is consistent with microarray indications that the *PRKAR1A *regulatory subunit of PKA was under-expressed in high-lonely participants, which could constitutively de-repress PKA signaling to CREB. Decreased activity of Oct transcription factors is consistent with the observed under-expression of the Ikaros transcription factor (*ZNFN1A1*) and other B lymphocyte maturation markers.

**Table 3:** Transcription factor-binding motifs in promoters of loneliness-related genes

TFBM	Low lonely	High lonely	Ratio	*t*	*df*	*p*
V$CEBP_Q2 (C/EBP)	0.714	0.340	0.476	-2.76	120.3	0.0067
V$OCT1_03 (Oct)	1.400	0.793	0.566	-2.60	115.7	0.0106
V$CREL_01 (NF-κB)	0.129	0.377	2.935	2.62	74.6	0.0108
V$GATA1_03 (GATA)	0.557	0.245	0.440	-2.52	120.2	0.0131
V$OCT1_04 (Oct)	0.086	0.000	0.000	-2.54	69.0	0.0132
V$OCT_C (Oct)	0.071	0.000	0.000	-2.30	69.0	0.0242
V$AHRARNT_01 (AHR)	0.043	0.208	4.843	2.28	65.4	0.0258
V$BARBIE_01 (BARBIE)	0.043	0.208	4.843	2.28	65.4	0.0258
V$GR_Q6 (GRE)	0.257	0.094	0.367	-2.17	112.1	0.0325
V$STAT_01 (STAT)	0.014	0.151	10.566	2.13	57.4	0.0376
V$TAL1BETAE47_01	0.057	0.000	0.000	-2.04	69.0	0.0447
V$TAL1ALPHAE47_01	0.057	0.000	0.000	-2.04	69.0	0.0447
V$IRF1_01 (IRF1)	0.086	0.000	0.000	-1.93	69.0	0.0572

TFBM: transcription factor-binding motif (V$ = TRANSFAC Vertebrate). Low lonely: average TFBM prevalence in promoters of genes up-regulated in low-lonely. High lonely: average TFBM prevalence in promoters of genes up-regulated in high-lonely. Ratio: high-lonely/low-lonely. *t *Statistic: Welch formula for unequal variances. *df*: continuous degrees of freedom for Welch unequal variance *t*-test. *p *value: two-tailed.

TELiS analysis utilizing default optimal parameter settings (Table 3) also identified several other signaling alterations associated with social isolation, including: activation of STAT family transcription factors that mediate signaling through multiple cytokine and growth factor receptors; reduced activity of IRF1, which responds specifically to type I interferons (consistent with the previously noted down-regulation of interferon response genes); and reduced activity of GATA family transcription factors. These results emerged as statistically significant in primary analyes, but were sporadically non-significant when TELiS analytic parameters were parametrically varied [41]. Indications of STAT, IRF1, and GATA alterations should, therefore, be considered provisional. Among 192 vertebrate transcription control motifs analyzed by TELiS, no others showed consistent differential activity in association with subjective social isolation.

## Discussion

This study provides the first systematic analysis of genome-wide transcriptional alterations as a potential mechanism of social-epidemiological influences on human health. Individuals who experience themselves as chronically isolated from others have an increased risk of several inflammation-related diseases [1-6], and the broad pattern of leukocyte transcriptional alterations identified in this study provides a framework for understanding that risk at the molecular level. Immune cells from people who report consistently high levels of subjective social isolation (loneliness) showed increased expression of genes controlling basic cellular transcription processes, cell cycle progression, pro-inflammatory cytokine signaling, and prostaglandin synthesis. Against this generalized backdrop of immunological activation, however, several functionally distinct subsets of immune response genes showed selective under-expression, including type I interferon response genes involved in innate antiviral resistance, and genes supporting antibody production and mature B lymphocyte function. These leukocyte transcriptional dynamics are consistent with clinical data indicating a complex pattern of host resistance alterations in social isolates, including increased risk of inflammation-mediated disease [1,2], accompanied by decreased resistance to viral infection [3,4,16,25] and impaired humoral immune response [29]. In addition to providing a molecular framework for understanding the biological mechanisms of social epidemiology [48,49], the present results provide a functional genomic target for the rational selection of biological interventions to remediate those effects. The transcriptional fingerprint of loneliness identified here may also provide a novel genomic biomarker for assessing the impact of such interventions prior to the onset of clinical disease.

A key contribution of the present study involves the use of recently developed structure/function bioinformatics to identify candidate upstream transcription control pathways that drive *in vivo *functional genomic alterations associated with social risk factors. Decreased activity of the anti-inflammatory GR pathway and reciprocal increases in NF-κB/Rel and JAK/STAT signaling represent plausible mediators of the increased immune activation profile observed in this study at the level of the leukocyte transcriptome. Reduced expression of GR-responsive genes is particularly remarkable in light of the fact that high-lonely individuals showed circulating cortisol levels that were broadly comparable to those of socially integrated individuals, and slightly higher during the late-day nadir in hypothalamic-pituitary-adrenal (HPA) axis output (consistent with previous findings) [27-30]. Thus, reduced expression of GR target genes in leukocytes from high-lonely individuals does not appear to stem from a failure of the HPA axis to maintain normal circulating cortisol levels, but rather from a reduction in GR-mediated transduction of that hormonal signal into the cellular transcriptome. This hypothesis is consistent with previous indications of receptor-mediated glucocorticoid insensitivity during behavioral stress [38,40], and with recently identified molecular mechanisms of GR transcriptional inhibition [37]. Loneliness was not associated with a reduction in GR (*NR3C1*) mRNA levels, suggesting that post-transcriptional modification of the GR is the most likely mechanism of transcriptional inhibition [37]. Further analyses will be required to identify the specific molecular locus of GR desensitization, but the broad alterations in inflammatory gene expression identified in the present study suggest that the GR's functional modification has physiological significance at the level of *in vivo *gene regulation. Thus, human genomic function is indeed sensitive to social environmental conditions, but transcriptional alterations may not always track alterations in neuroendocrine activity due to intervening variations in the activity of signal transduction pathways (for example, GR insensitivity).

This study has identified a clear genomic fingerprint of social isolation, and defined candidate transcription control pathways that may shape its expression, but several limitations must be considered when interpreting these results. First, the present findings are based on a relatively small number of individuals sampled from the low and high extremes of a social-epidemiological risk dimension, and thus require replication in larger samples that are more broadly representative of the total variation in human social phenotypes. However, it is remarkable that the size and inter-individual consistency of transcriptional alterations associated with subjective social isolation is sufficiently pronounced to reach high levels of statistical significance in a relatively small sample. It is unclear whether alterations in inflammatory signaling and gene expression would occur in other cell types besides leukocytes, or whether different patterns of transcriptional alteration might be observed in other tissues. It is also unclear whether the strong quantitative relationship between subjective social isolation and leukocyte transcriptional profiles observed here stems from a causal effect of social processes on gene expression (for example, via the neuroendocrine system), or whether differential gene expression in the immune system might instead drive variations in social behavior (for example, via effects of pro-inflammatory cytokines and prostaglandins on central nervous system function) [50]. The effects of acute 'sickness behavior' are unlikely to explain the present results because this study analyzed long-term individual differences in experienced loneliness (that is, consistently expressed over at least three years). However, longitudinal studies will be required to rule out the possibility that variations in chronic inflammation might potentially influence long-term individual differences in social behavior. This study's identification of a plausible neuroendocrine mediator of transcriptional alteration (GR signaling), which is known to relate to social phenotype in humans [27-30] and is causally impacted by experimental social isolation in animal models [11-14], is consistent with social influences on gene expression. Experimental manipulations of long term social behavior may ultimately be required to definitively establish causation in the human clinical setting. What is clear from this study's ancillary analyses is that relationships between gene expression and social isolation cannot be attributed to correlated differences in other known demographic, psychological, social, or medical risk factors (including perceived stress, depression, hostility, socio-economic status, and altered subset distributions within the circulating leukocyte pool). Ancillary analyses controlling for transient variations in loneliness also suggest that the transcriptional correlates of chronic subjective social isolation (trait loneliness) do not stem solely from transient variations in state loneliness. A persistent sense of social isolation appears to represent a distinct epidemiological risk factor that is associated with broad alterations in immune cell gene expression linked to reciprocal shifts in the activity of pro- and anti-inflammatory transcription control pathways.

There is controversy in the social epidemiology literature about whether the health risks of social isolation stem mainly from an objective lack of social contact (for example, diminishing physical, cognitive, or economic assistance) or from the subjective experience of social isolation (leading to perceptions of threat/uncertainty that activate neuroendocrine stress responses) [2,28,51]. In the present study, the functional genomic correlates of subjective social isolation were found to be largely independent of the objective size of an individual's social network. This result underscores the key role of subjective perceptual processes in transmitting the effects of social factors into physical biology via neuroendocrine alterations, and their subsequent impact on cellular gene expression [4,28,51,52]. Moreover, bioinformatics analyses identified several candidate transcription control pathways that could plausibly mediate those effects, including the inflammation-related GR, NF-κB/Rel, and JAK/STAT pathways, as well as the CREB/ATF, IRF1, GATA, and Oct families of transcription factors. These candidate 'social signal transduction pathways' provide a mechanistic basis for understanding social epidemiology in terms of molecular interactions between the human genome and the external socio-environmental stimuli that regulate cellular gene expression [26,48,49].

## Conclusion

These data identify a distinct transcriptional fingerprint of subjective social isolation in human leukocytes, which involves increased basal expression of inflammatory and immune response genes. Bioinformatic analysis of differentially expressed promoters suggests that these effects may be shaped by reduced activity of the anti-inflammatory glucocorticoid transcription control pathway and a complementary increase in activity of the pro-inflammatory NF-κB/Rel pathway. These data provide the first evidence that social-environmental risk factors are linked to global alterations in human gene transcription, and they establish a molecular context for understanding the increased risk of inflammatory disease observed in human beings who experience a chronic sense of subjective social isolation (loneliness). Dissociation between stable circulating cortisol levels and impaired glucocorticoid receptor-mediated transcription highlights the need to analyze social environmental risk at the level of the functional genomic dynamics that ultimately drive the expression of disease.

## Materials and methods

### Participants

Data come from the CHASRS - a 5-year cohort analysis of psychological, social, economic, and biomedical outcomes in 230 English-speaking men and women aged 50-67 years at study entry [43]. Annual study visits collected detailed biomedical, social, psychological, and economic assessments, including full medical histories, body mass index and waist circumference, diurnal measures of salivary cortisol, laboratory measures of autonomic nervous system activity, and psychometric measures of social network characteristics, stress, depression, and personality (detailed below). Health-relevant behavioral characteristics assessed include sleep quality, nutrition, exercise, memory and cognitive function. In 2005, 153 of the 166 remaining participants (92%) agreed to provide a 10 ml sample of peripheral blood for gene expression analysis. Gene expression analyses were carried out on samples from 14 individuals as outlined below. Procedures were approved by Institutional Review Boards at the University of Chicago and UCLA.

### Social isolation

Subjectively experienced social isolation was assessed by the UCLA-R Loneliness scale [53] at each yearly study visit. Biological samples from 10 individuals who consistently scored in the top 15% of the loneliness distribution during study years 1, 2, and 3, and 10 individuals who consistently scored in the bottom 15% during years 1, 2, and 3, were selected for analysis after matching for age, gender, and ethnicity. Two samples from low-lonely individuals and four samples from high-lonely individuals yielded insufficient RNA for reliable gene expression assay, and analyses are thus based on fourteen individuals (eight low-lonely, six high-lonely). Objective social isolation was assessed by the social network index [54].

### Background characteristics and confounders

Demographic, social, psychological, and medical characteristics were assessed using established instruments [43], including measures of depression [55], perceived stress [56], hostility [57], body mass index, physician-diagnosed medical conditions, use of anti-inflammatory agents (including non-steroidal anti-inflammatories), diuretics, statins, beta-blockers, anti-diabetic agents, and anti-depressants. Participants were free of infectious disease symptoms, major medical conditions, and anti-inflammatory medications at the time of data collection. Samples were collected between 10 am and 2 pm (median = 11 am for both groups).

### Leukocyte gene expression

Total RNA was extracted from 10^7 ^circulating leukocytes (mononuclear cells collected from a 10 ml antecubital venipuncture sample and isolated by ficoll density gradient centrifugation; RNAlater/RNeasy, Qiagen, Valencia, CA, USA), and 5 μg of the resulting RNA was assayed using Affymetrix U133A high-density oligonucleotide arrays [58] in the UCLA DNA Microarray Core as previously described [41,59]. Robust multiarray averaging [60] was applied to quantify expression of the 22,283 assayed transcripts, and differentially expressed genes were identified as those showing ≥30% difference in mean expression levels in samples from high- versus low-lonely individuals (corresponding to a FDR of 10%) [59]. Functional characteristics of individual genes were identified through GO annotations, Gene References into Function annotations, and PubMed literature links retrieved through NCBI Entrez-Gene [61]. Functional commonalities among multiple differentially expressed genes were identified using GOstat [62] with default stringency parameters (Benjamini FDR <0.10) [44]. A full list of differentially expressed genes is provided in Additional data file 1, and raw data are deposited in the National Center for Biotechnology Information Gene Expression Omnibus (GEO Series GSE7148).

### RT-PCR

Transcripts identified as differentially expressed in microarray analyses were independently assayed by quantitative real-time RT-PCR using TaqMan Gene Expression Assays (Applied Biosystems, Foster City, CA, USA): *IL8 *(Hs00174103_m1), *CDC25B *(Hs00244740_m1), *IL1B *(Hs00174097_m1), *EGR1 *(Hs00152928_m1), *FOSB *(Hs01547109_m1), *PTGS2 *(Hs00153133_m1), *IFI6/G1P3 *(Hs00242571_m1), *GAPDH *(Hs99999905_m1). We also verified microarray indications of equivalent expression of GR mRNA, *NR3C1 *(Hs00353740_m1). Assays for each sample (*n *= 14) were carried out in triplicate using an iCycler instrument (Biorad, Hercules, CA, USA), Quantitect Probe RT-PCR enzymes (Qiagen), and the manufacturer's recommended 1-step thermal cycling protocol. *ISGF12 *was assayed using published primer sequences and reaction conditions [33]. Threshold cycle numbers for each analyte were normalized to GAPDH for analysis. A multivariate analysis of variance (MANOVA) tested group differences among all analytes simultaneously, and independent sample *t*-tests assessed differential expression of each individual transcript.

### Transcriptional mediation analysis

A 2-sample variant of TELiS [63] identified upstream signal transduction pathways driving differential gene expression [41]. Primary analyses compared the prevalence of vertebrate V$GR_Q6 and V$CREL_01 TFBMs from the TRANSFAC database [64] in promoters of genes over-expressed in high- versus low-lonely individuals. Primary analyses utilized default scan parameters [41] and identified differences in TFBM prevalence using *p *values from an independent sample *t*-test with Welch's correction for heteroscedasticity [65]. To assess the robustness of results to technical variations, sensitivity analyses examined parametric variations of promoter length (-300 bp relative to RefSeq transcription start site, -600 bp, and -1,000 bp to +200), and motif match stringency (MatSim = 0.80, 0.90, 0.95) [41]. Sensitivity analyses also tested the impact of increasing stringency in the definition of differential gene expression (capture based on 1.5-fold change, corresponding to 5% FDR) [59].

Ancillary analyses utilized ANCOVA [65] to control for potential confounding between subjective social isolation (denoted *x*) and other demographic, psychological, or medical covariates (denoted *c*) that might influence gene expression. In these analyses, the profile of expression of each gene across participants (denoted *y*) was first adjusted to remove any variance attributable to a covariate (based on the general linear model *y *= *c *+ *e*), and the residual covariate-adjusted gene expression profile (*e*) was then tested for significant difference as a function of social isolation (that is, in the general linear model *e *= *x *+ *z*, where *z *represents residual error) [65]. Genes identified as differentially expressed based on analysis of covariate-adjusted expression profiles were then analyzed as described above for differential prevalence of promoter TFBMs.

Reciprocal activity of NF-κB response elements and GREs was quantified by log-transformed prevalence ratios (NF-κB TFBM prevalence/GRE TFBM prevalence) in promoters of differentially expressed genes. Welch *t*-tests evaluated statistical significance of reciprocal bias (log ratio ≠ 0), and a Monte Carlo random sampling analysis assessed the probability that observed biases stemmed from an inverse relationship between GRE and NF-κB response elements within the *cis*-structure of the human promoter population (rather than the *trans*-activational activity targeted by TELiS). Monte Carlo analysis involved 10,000 cycles in which: 1.) a random set of genes equivalent in number to those found to be differentially expressed in socially isolated versus integrated individuals were sampled from the population of human genes assayed by the Affymetrix U133A array; and 2.) the NF-κB/GRE prevalence ratio was calculated for each random sample. The resulting sampling distribution of 10,000 null hypothesis ratios quantified the probability that a random set of human genes would generate a NF-κB/GRE prevalence ratio greater than or equal to that shown by the observed set of differentially expressed genes.

Following hypothesis-driven analyses of NF-κB/Rel and GR activity, additional exploratory analyses analyzed the prevalence of 190 other vertebrate TFBMs in the TELiS database [41], including parametric variations of promoter length and motif match stringency to evaluate the sensitivity of results to analytical parameters.

### Glucocorticoid level

HPA axis activity was assessed by radioimmunoassay of cortisol concentrations in saliva samples collected immediately on waking (0 h), 30 minutes later (0.5 h), and immediately prior to evening repose (16 h), on 3 consecutive days during each of 3 study years [27]. Log-transformed cortisol concentrations were analyzed using mixed effect linear models (SAS PROC MIXED; SAS Institute, Cary, NC, USA) treating time of day as a 3-level repeated-measures factor or a linear effect in hours post-awakening (0, 0.5, 16). Day (1-3) and year (1-4) were modeled as repeated measures, and individual differences in cortisol level and diurnal decline were modeled as a random effects nested within low- versus high-loneliness.

### C-reactive protein

Systemic inflammatory biology was assessed by circulating levels of CRP. Blood spot samples obtained during study years 2, 3, and 4 were analyzed using a high-sensitivity enzyme immunoassay [66], and longitudinal observations on each participant were analyzed using a mixed effect linear model (SAS PROC MIXED) in a Year (2, 3, and 4, treated as a repeated measure) × Loneliness (high versus low) factorial design. Aberrantly high CRP values were identified by standard outlier detection algorithms (SAS PROC UNIVARIATE) and excluded from analysis because they were likely to reflect the effect of acute infection rather than chronic inflammation.

## Abbreviations

ANCOVA, analysis of covariance; CHASRS, Chicago Health Aging and Social Relations Study; CRP, C-reactive protein; FDR, false discovery rate; GO, Gene Ontology; GR, glucocorticoid receptor; GRE, glucocorticoid response element; HPA, hypothalamic-pituitary-adrenal; IRF, interferon response factor; JAK/STAT, Janus kinase-signal transducer and activator of transcription; NF, nuclear factor; TELiS, Transcription Element Listening System; TFBM, transcription factor-binding motif.

## Authors' contributions

SW Cole: Study conception, research design, data collection, data analysis, manuscript production.

LC Hawkley: Data collection, data analysis, manuscript production.

JM Arevalo: Data collection and assays, manuscript production.

CY Sung: Data collection and assays, manuscript production.

RM Rose: Study conception, manuscript production.

JT Cacioppo: Study conception, research design, data collection, data analysis, manuscript production.

## Additional data files

The following additional data are available with the online version of this paper. Additional data file 1 is a table listing transcripts identified by microarray analysis as differentially expressed in leukocytes from high- versus low-lonely individuals. Additional data file 2 is a figure presenting results of confirmatory RT-PCR analyses verifying differential expression of selected transcripts.

## Supplementary Material

Additional data file 1 Transcripts identified by microarray analysis as differentially expressed in leukocytes from high- versus low-lonely individuals. Click here for file

Additional data file 2 Results of confirmatory RT-PCR analyses verifying differential expression of selected transcripts. Click here for file
