# Supplementary material for: Social regulation of gene expression in human leukocytes
Source: Genome Biol. 2007 Sep 13;8(9):R189. doi: 10.1186/gb-2007-8-9-r189 (PMC2375027; doi:10.1186/gb-2007-8-9-r189)
Supplement: Additional data file 1 — Transcripts identified by microarray analysis as differentially expressed in leukocytes from high- versus low-lonely individuals. [file gb-2007-8-9-r189-S1.doc]

**ADF1. Genes differentially expressed in individuals experiencing high vs. low levels of subjective social isolation.**

| **Affymetrix Probe Name** | **Gene Symbol** | **Mean**^1^ | | **SD** | | **Ratio**^2^ | **r**^3^ |
| --- | --- | --- | --- | --- | --- | --- | --- |
|  |  | Low-Lonely | High-Lonely | Low-Lonely | High-Lonely |  |  |
| **Genes over-expressed in High-Lonely** | | | | | | | |
| 220532_s_at | *LR8* | 7.73 | 9.26 | 1.37 | 0.79 | 2.89 | 0.404 |
| 221491_x_at | Unnamed transcript | 7.34 | 8.77 | 1.12 | 0.48 | 2.68 | 0.717 |
| 201694_s_at | *EGR1* | 6.35 | 7.62 | 0.58 | 1.57 | 2.40 | 0.470 |
| 218345_at | *HCA112* | 7.52 | 8.78 | 0.98 | 0.78 | 2.40 | 0.429 |
| 208151_x_at | *DDX17* | 6.74 | 7.85 | 0.62 | 1.05 | 2.15 | 0.609 |
| 208719_s_at | *DDX17* | 5.83 | 6.82 | 0.57 | 1.06 | 1.99 | 0.564 |
| 202859_x_at | *IL8* | 7.17 | 8.14 | 1.56 | 1.86 | 1.95 | 0.360 |
| 201693_s_at | *EGR1* | 4.99 | 5.89 | 0.17 | 1.42 | 1.87 | 0.359 |
| 203290_at | *HLA-DQA1* | 6.77 | 7.67 | 1.98 | 1.50 | 1.87 | 0.337 |
| 209156_s_at | *COL6A2* | 5.86 | 6.63 | 0.41 | 0.66 | 1.70 | 0.484 |
| 210095_s_at | *IGFBP3* | 6.28 | 6.97 | 0.57 | 0.67 | 1.61 | 0.403 |
| 205114_s_at | *MGC12815* | 6.93 | 7.61 | 0.64 | 0.83 | 1.60 | 0.541 |
| 213524_s_at | *G0S2* | 6.19 | 6.85 | 1.12 | 2.12 | 1.58 | 0.209 |
| 218136_s_at | *MSCP* | 5.63 | 6.27 | 0.48 | 1.06 | 1.56 | 0.470 |
| 202768_at | *FOSB* | 5.11 | 5.75 | 0.28 | 0.65 | 1.56 | 0.526 |
| 204748_at | *PTGS2* | 4.79 | 5.42 | 0.62 | 1.80 | 1.56 | 0.145 |
| 211506_s_at | *IL8* | 5.47 | 6.11 | 1.15 | 2.01 | 1.56 | 0.157 |
| 209788_s_at | *ARTS-1* | 7.74 | 8.28 | 0.32 | 0.45 | 1.46 | 0.570 |
| 206115_at | *EGR3* | 4.06 | 4.60 | 0.15 | 1.15 | 1.46 | 0.187 |
| 201082_s_at | *DCTN1* | 5.38 | 5.89 | 0.30 | 0.49 | 1.43 | 0.309 |
| 215555_at | Unnamed transcript | 4.26 | 4.76 | 0.33 | 0.18 | 1.42 | 0.653 |
| 213998_s_at | *DDX17* | 6.71 | 7.22 | 0.34 | 0.82 | 1.42 | 0.365 |
| 214218_s_at | *XIST* | 7.87 | 8.37 | 2.57 | 1.95 | 1.42 | 0.173 |
| 39402_at | *IL1B* | 5.45 | 5.95 | 0.23 | 0.83 | 1.41 | 0.301 |
| 210119_at | *KCNJ15* | 4.28 | 4.78 | 0.23 | 0.96 | 1.41 | 0.189 |
| 203231_s_at | *ATXN1* | 5.52 | 5.99 | 0.48 | 0.90 | 1.39 | 0.388 |
| 207723_s_at | *KLRC3* | 5.98 | 6.45 | 0.56 | 0.69 | 1.39 | 0.409 |
| 218164_at | *SSP411* | 6.62 | 7.09 | 0.45 | 0.21 | 1.39 | 0.227 |
| 213906_at | *MYBL1* | 8.09 | 8.55 | 0.57 | 0.78 | 1.38 | 0.277 |
| 207008_at | *IL8RB* | 6.00 | 6.45 | 0.19 | 0.89 | 1.37 | 0.166 |
| 220110_s_at | *NXF3* | 4.27 | 4.73 | 0.13 | 1.06 | 1.37 | 0.439 |
| 221920_s_at | *MSCP* | 5.26 | 5.70 | 0.65 | 1.09 | 1.36 | 0.318 |
| 203200_s_at | *MTRR* | 7.52 | 7.96 | 0.24 | 0.36 | 1.36 | 0.397 |
| 215666_at | *HLA-DRB4* | 4.34 | 4.77 | 0.81 | 1.27 | 1.35 | 0.087 |
| 205067_at | *IL1B* | 6.53 | 6.96 | 0.20 | 0.91 | 1.35 | 0.224 |
| 216050_at | Unnamed transcript | 4.40 | 4.82 | 0.47 | 1.06 | 1.34 | 0.255 |
| 204411_at | *KIF21B* | 7.69 | 8.12 | 0.20 | 0.33 | 1.34 | 0.553 |
| 221728_x_at | *XIST* | 7.95 | 8.38 | 2.19 | 1.99 | 1.34 | 0.162 |
| 201908_at | *DVL3* | 5.68 | 6.10 | 0.18 | 0.25 | 1.34 | 0.582 |
| 209433_s_at | *PPAT* | 5.37 | 5.79 | 0.22 | 0.28 | 1.34 | 0.604 |
| 204794_at | *DUSP2* | 6.78 | 7.19 | 0.30 | 0.60 | 1.33 | 0.389 |
| 201074_at | *SMARCC1* | 7.06 | 7.47 | 0.30 | 0.31 | 1.33 | 0.437 |
| 203668_at | *MAN2C1* | 6.51 | 6.91 | 0.26 | 0.21 | 1.33 | 0.556 |
| 215147_at | Unnamed transcript | 6.37 | 6.78 | 0.46 | 0.44 | 1.33 | 0.421 |
| 216834_at | *RGS1* | 4.31 | 4.72 | 0.42 | 0.42 | 1.33 | 0.685 |
| 210649_s_at | *ARID1A* | 6.89 | 7.30 | 0.27 | 0.40 | 1.33 | 0.445 |
| 206666_at | *GZMK* | 10.37 | 10.78 | 0.42 | 0.50 | 1.33 | 0.521 |
| 215894_at | *PTGDR* | 5.44 | 5.85 | 0.37 | 0.93 | 1.33 | 0.260 |
| 212205_at | *H2AFV* | 8.05 | 8.46 | 0.30 | 0.24 | 1.33 | 0.646 |
| 201236_s_at | *BTG2* | 8.67 | 9.07 | 0.25 | 0.63 | 1.32 | 0.301 |
| 210425_x_at | Unnamed transcript | 8.56 | 8.96 | 0.42 | 0.24 | 1.32 | 0.441 |
| 212375_at | *EP400* | 6.85 | 7.25 | 0.14 | 0.34 | 1.32 | 0.416 |
| 212152_x_at | *ARID1A* | 8.67 | 9.08 | 0.15 | 0.28 | 1.32 | 0.527 |
| 204912_at | *IL10RA* | 9.81 | 10.21 | 0.36 | 0.32 | 1.32 | 0.473 |
| 221860_at | *HNRPL* | 7.34 | 7.73 | 0.37 | 0.49 | 1.32 | 0.299 |
| 215470_at | Unnamed transcript | 4.60 | 5.00 | 0.18 | 0.38 | 1.32 | 0.482 |
| 212993_at | Unnamed transcript | 6.90 | 7.30 | 0.31 | 0.29 | 1.32 | 0.361 |
| 204670_x_at | *HLA-DRB* | 11.80 | 12.19 | 0.34 | 0.16 | 1.32 | 0.468 |
| 202423_at | *MYST3* | 9.16 | 9.56 | 0.30 | 0.35 | 1.31 | 0.482 |
| 221768_at | *SFPQ* | 6.15 | 6.54 | 0.46 | 0.56 | 1.31 | 0.280 |
| 201392_s_at | *IGF2R* | 6.65 | 7.03 | 0.29 | 0.25 | 1.31 | 0.442 |
| 206366_x_at | *XCL2* | 8.47 | 8.86 | 0.40 | 0.80 | 1.31 | 0.367 |
| 218066_at | *SLC12A7* | 8.49 | 8.88 | 0.41 | 0.38 | 1.31 | 0.427 |
| 202379_s_at | *NKTR* | 8.57 | 8.96 | 0.46 | 0.57 | 1.31 | 0.260 |
| 203691_at | *PI3* | 5.18 | 5.57 | 0.12 | 0.84 | 1.31 | 0.298 |
| 205180_s_at | *ADAM8* | 8.12 | 8.51 | 0.17 | 0.25 | 1.30 | 0.514 |
| 211987_at | *TOP2B* | 8.73 | 9.12 | 0.26 | 0.23 | 1.30 | 0.600 |
| 208798_x_at | *GOLGIN-67* | 7.85 | 8.23 | 0.41 | 0.30 | 1.30 | 0.274 |
| 210746_s_at | *EPB42* | 5.66 | 6.04 | 1.02 | 2.02 | 1.30 | 0.269 |
| 218098_at | *ARFGEF2* | 6.61 | 6.99 | 0.32 | 0.38 | 1.30 | 0.444 |
| 205230_at | *RPH3A* | 5.68 | 6.06 | 0.40 | 0.50 | 1.30 | 0.155 |
| 202081_at | *IER2* | 10.57 | 10.95 | 0.20 | 0.49 | 1.30 | 0.591 |
| 215819_s_at | *RHCE* | 5.35 | 5.73 | 0.14 | 0.57 | 1.30 | 0.588 |
| 201853_s_at | *CDC25B* | 9.17 | 9.55 | 0.25 | 0.32 | 1.30 | 0.505 |
| 210070_s_at | *CPT1B* | 5.52 | 5.90 | 0.33 | 0.25 | 1.30 | 0.483 |
| 209728_at | *HLA-DRB4* | 8.34 | 8.71 | 2.62 | 2.48 | 1.30 | -0.035 |
| 202644_s_at | *TNFAIP3* | 8.66 | 9.04 | 0.16 | 0.33 | 1.30 | 0.487 |
| 201170_s_at | *BHLHB2* | 7.88 | 8.26 | 0.43 | 0.28 | 1.30 | 0.371 |
| **Genes over-expressed in Low-Lonely (under-expressed in High-Lonely)** | | | | | | | |
| 219449_s_at | *FLJ20533* | 8.53 | 8.03 | 0.42 | 0.28 | 0.70 | -0.554 |
| 213348_at | *CDKN1C* | 7.57 | 7.06 | 0.62 | 0.91 | 0.70 | -0.324 |
| 206788_s_at | *CBFB* | 7.70 | 7.19 | 0.38 | 0.59 | 0.70 | -0.297 |
| 221638_s_at | *STX16* | 5.90 | 5.39 | 0.30 | 0.53 | 0.70 | -0.375 |
| 210734_x_at | *MAX* | 8.43 | 7.92 | 0.36 | 0.45 | 0.70 | -0.492 |
| 208791_at | *CLU* | 8.87 | 8.35 | 0.46 | 1.20 | 0.70 | -0.298 |
| 207791_s_at | *RAB1A* | 7.87 | 7.36 | 0.37 | 0.33 | 0.70 | -0.415 |
| 200778_s_at | *NEDD5* | 8.58 | 8.06 | 0.31 | 0.55 | 0.70 | -0.390 |
| 208579_x_at | *HIST1H2BK* | 8.79 | 8.27 | 0.57 | 0.46 | 0.70 | -0.366 |
| 206871_at | *ELA2* | 6.09 | 5.57 | 1.00 | 0.91 | 0.70 | -0.042 |
| 221651_x_at | *IGKC* | 12.33 | 11.81 | 0.66 | 0.50 | 0.70 | -0.401 |
| 212827_at | *IGHM* | 9.83 | 9.31 | 0.64 | 1.16 | 0.70 | -0.452 |
| 206390_x_at | *PF4* | 11.30 | 10.78 | 0.32 | 1.08 | 0.70 | -0.248 |
| 201559_s_at | *CLIC4* | 5.78 | 5.25 | 0.46 | 0.35 | 0.70 | -0.472 |
| 200604_s_at | *PRKAR1A* | 8.80 | 8.27 | 0.33 | 0.44 | 0.69 | -0.416 |
| 214544_s_at | *SNAP23* | 7.85 | 7.32 | 0.33 | 0.51 | 0.69 | -0.394 |
| 218999_at | *FLJ11000* | 7.98 | 7.45 | 0.34 | 0.50 | 0.69 | -0.424 |
| 205221_at | *HGD* | 6.14 | 5.60 | 0.85 | 0.33 | 0.69 | -0.458 |
| 205297_s_at | *CD79B* | 8.21 | 7.67 | 0.66 | 0.69 | 0.69 | -0.470 |
| 214196_s_at | *CLN2* | 8.56 | 8.01 | 0.39 | 0.36 | 0.69 | -0.456 |
| 214146_s_at | *PPBP* | 11.63 | 11.09 | 0.22 | 0.90 | 0.69 | -0.311 |
| 201059_at | *CTTN* | 6.79 | 6.25 | 0.35 | 0.60 | 0.69 | -0.460 |
| 221671_x_at | *IGKC* | 12.22 | 11.67 | 0.63 | 0.48 | 0.69 | -0.438 |
| 205513_at | *TCN1* | 7.20 | 6.66 | 0.87 | 1.14 | 0.68 | -0.017 |
| 217235_x_at | Unnamed transcript | 7.82 | 7.27 | 0.71 | 0.26 | 0.68 | -0.436 |
| 219607_s_at | *MS4A4A* | 5.19 | 4.64 | 0.78 | 0.49 | 0.68 | -0.237 |
| 215071_s_at | *HIST1H2AC* | 9.25 | 8.70 | 0.69 | 0.74 | 0.68 | -0.354 |
| 208653_s_at | *CD164* | 7.42 | 6.87 | 0.33 | 0.58 | 0.68 | -0.293 |
| 210356_x_at | *MS4A1* | 9.27 | 8.71 | 0.77 | 0.88 | 0.68 | -0.451 |
| 215946_x_at | *IGLL1* | 8.21 | 7.65 | 0.61 | 0.31 | 0.68 | -0.334 |
| 207794_at | *CCR2* | 8.13 | 7.57 | 0.79 | 0.58 | 0.68 | -0.352 |
| 202687_s_at | *TNFSF10* | 8.59 | 8.02 | 0.54 | 0.41 | 0.68 | -0.255 |
| 213470_s_at | *HNRPH1* | 6.96 | 6.39 | 0.31 | 0.43 | 0.68 | -0.582 |
| 208490_x_at | *HIST1H2BG* | 7.04 | 6.47 | 0.51 | 0.44 | 0.67 | -0.460 |
| 205844_at | *VNN1* | 5.90 | 5.33 | 0.65 | 0.56 | 0.67 | -0.414 |
| 209257_s_at | *CSPG6* | 7.24 | 6.67 | 0.27 | 0.42 | 0.67 | -0.546 |
| 211654_x_at | *HLA-DQB1* | 10.17 | 9.60 | 0.65 | 0.78 | 0.67 | -0.290 |
| 214973_x_at | Unnamed transcript | 7.55 | 6.98 | 0.79 | 0.37 | 0.67 | -0.351 |
| 204415_at | *G1P3* | 8.00 | 7.42 | 0.88 | 0.40 | 0.67 | -0.153 |
| 206133_at | *HSXIAPAF1* | 8.35 | 7.78 | 0.83 | 0.24 | 0.67 | -0.047 |
| M97935_5_at | *STAT1* | 6.26 | 5.68 | 0.61 | 0.74 | 0.67 | -0.074 |
| 219534_x_at | *CDKN1C* | 6.85 | 6.27 | 0.49 | 0.68 | 0.67 | -0.434 |
| 204860_s_at | *BIRC1* | 6.96 | 6.38 | 0.63 | 0.38 | 0.67 | -0.330 |
| 205267_at | *POU2AF1* | 8.29 | 7.71 | 0.66 | 0.96 | 0.67 | -0.475 |
| 217258_x_at | Unnamed transcript | 6.53 | 5.95 | 0.58 | 0.20 | 0.67 | -0.437 |
| 201120_s_at | *PGRMC1* | 8.16 | 7.58 | 0.45 | 0.59 | 0.67 | -0.403 |
| 214836_x_at | Unnamed transcript | 8.99 | 8.41 | 0.54 | 0.46 | 0.67 | -0.535 |
| 217418_x_at | *MS4A1* | 9.15 | 8.56 | 0.79 | 0.96 | 0.67 | -0.455 |
| 211650_x_at | *IGHG1* | 6.56 | 5.97 | 0.42 | 0.43 | 0.67 | -0.618 |
| 211635_x_at | Unnamed transcript | 7.48 | 6.89 | 0.50 | 0.47 | 0.66 | -0.575 |
| 215111_s_at | *TGFB1I4* | 8.26 | 7.67 | 0.55 | 0.30 | 0.66 | -0.523 |
| 216901_s_at | *ZNFN1A1* | 6.53 | 5.93 | 0.39 | 0.54 | 0.66 | -0.419 |
| 216915_s_at | *PTPN12* | 6.35 | 5.74 | 0.53 | 0.61 | 0.66 | -0.482 |
| 213872_at | Unnamed transcript | 9.63 | 9.02 | 0.45 | 0.54 | 0.65 | -0.402 |
| 211634_x_at | *IGHM* | 7.47 | 6.85 | 0.60 | 0.47 | 0.65 | -0.479 |
| 215936_s_at | *KIAA1033* | 7.14 | 6.53 | 0.33 | 0.43 | 0.65 | -0.591 |
| 213674_x_at | *IGHM* | 8.49 | 7.87 | 0.68 | 1.27 | 0.65 | -0.467 |
| 211639_x_at | *IGHM* | 7.49 | 6.87 | 0.62 | 0.25 | 0.65 | -0.378 |
| 217179_x_at | Unnamed transcript | 7.12 | 6.50 | 0.58 | 0.44 | 0.65 | -0.462 |
| 209131_s_at | *SNAP23* | 7.26 | 6.64 | 0.39 | 0.57 | 0.65 | -0.477 |
| 200796_s_at | *MCL1* | 7.13 | 6.51 | 0.57 | 0.91 | 0.65 | -0.265 |
| 211798_x_at | *IGLJ3* | 7.54 | 6.91 | 0.58 | 0.47 | 0.65 | -0.549 |
| 205442_at | *MFAP3L* | 6.43 | 5.80 | 0.42 | 0.70 | 0.65 | -0.455 |
| 209374_s_at | *IGHM* | 10.66 | 10.02 | 0.51 | 0.95 | 0.64 | -0.531 |
| 208097_s_at | *TXNDC* | 7.95 | 7.32 | 0.35 | 0.48 | 0.64 | -0.489 |
| 214453_s_at | *IFI44* | 7.97 | 7.33 | 1.14 | 0.53 | 0.64 | -0.061 |
| 211881_x_at | *IGLJ3* | 7.31 | 6.67 | 0.58 | 0.46 | 0.64 | -0.499 |
| 206108_s_at | *SFRS6* | 7.23 | 6.59 | 0.55 | 0.51 | 0.64 | -0.530 |
| 202869_at | *OAS1* | 8.34 | 7.69 | 0.87 | 0.45 | 0.64 | -0.148 |
| 209480_at | *HLA-DQB1* | 6.78 | 6.13 | 2.42 | 2.39 | 0.64 | 0.018 |
| 217232_x_at | Unnamed transcript | 13.37 | 12.72 | 0.31 | 1.61 | 0.64 | -0.265 |
| 214768_x_at | Unnamed transcript | 7.16 | 6.51 | 0.69 | 0.63 | 0.63 | -0.503 |
| 215118_s_at | *MGC27165* | 6.59 | 5.93 | 0.75 | 0.71 | 0.63 | -0.590 |
| 217281_x_at | *MGC27165* | 7.19 | 6.53 | 0.56 | 0.26 | 0.63 | -0.504 |
| 216984_x_at | Unnamed transcript | 7.44 | 6.77 | 0.73 | 0.43 | 0.63 | -0.433 |
| 216401_x_at | Unnamed transcript | 7.74 | 7.07 | 0.70 | 0.56 | 0.63 | -0.534 |
| 205483_s_at | *G1P2* | 9.46 | 8.79 | 0.89 | 0.46 | 0.63 | -0.057 |
| 213831_at | *HLA-DQA* | 6.99 | 6.32 | 2.32 | 2.50 | 0.63 | -0.001 |
| 208546_x_at | Unnamed transcript | 6.74 | 6.04 | 0.55 | 0.46 | 0.62 | -0.507 |
| 216576_x_at | Unnamed transcript | 7.63 | 6.92 | 0.71 | 0.61 | 0.61 | -0.473 |
| 202589_at | *TYMS* | 6.22 | 5.52 | 0.77 | 0.52 | 0.61 | -0.214 |
| 216207_x_at | *IGKV1D-13* | 8.88 | 8.17 | 0.68 | 0.59 | 0.61 | -0.476 |
| 202503_s_at | *KIAA0101* | 5.80 | 5.09 | 0.94 | 0.64 | 0.61 | -0.164 |
| 205552_s_at | *OAS1* | 7.20 | 6.48 | 0.83 | 0.40 | 0.61 | -0.220 |
| 215214_at | *IGLC2* | 6.65 | 5.93 | 0.77 | 0.32 | 0.61 | -0.486 |
| 214336_s_at | *COPA* | 6.63 | 5.91 | 0.44 | 0.55 | 0.61 | -0.434 |
| 206655_s_at | *GP1BB* | 8.26 | 7.54 | 0.58 | 0.99 | 0.61 | -0.391 |
| 214669_x_at | *IGKC* | 9.88 | 9.15 | 0.65 | 0.57 | 0.60 | -0.562 |
| 204560_at | *FKBP5* | 7.38 | 6.64 | 0.48 | 0.48 | 0.60 | -0.624 |
| 206110_at | *HIST1H3H* | 7.29 | 6.55 | 1.04 | 0.64 | 0.60 | -0.395 |
| 210732_s_at | *LGALS8* | 6.14 | 5.41 | 0.51 | 0.49 | 0.60 | -0.540 |
| 216510_x_at | *IGHG1* | 6.57 | 5.83 | 0.60 | 0.34 | 0.60 | -0.604 |
| 209773_s_at | *RRM2* | 5.91 | 5.17 | 1.20 | 0.44 | 0.60 | -0.191 |
| 200665_s_at | *SPARC* | 9.16 | 8.42 | 0.56 | 1.07 | 0.60 | -0.382 |
| 213797_at | *cig5* | 6.09 | 5.34 | 1.29 | 0.37 | 0.60 | -0.063 |
| 202411_at | *IFI27* | 5.69 | 4.92 | 2.08 | 0.23 | 0.59 | 0.058 |
| 217148_x_at | *IGLJ3 /// IGLC2* | 8.92 | 8.15 | 0.77 | 0.59 | 0.58 | -0.475 |
| 214777_at | Unnamed transcript | 7.57 | 6.79 | 0.64 | 0.25 | 0.58 | -0.573 |
| 203153_at | Unnamed transcript | 7.41 | 6.60 | 1.42 | 0.56 | 0.57 | -0.054 |
| 215379_x_at | *IGLC2* | 10.00 | 9.18 | 0.78 | 0.57 | 0.57 | -0.510 |
| 206641_at | *TNFRSF17* | 5.15 | 4.32 | 0.95 | 0.32 | 0.57 | -0.405 |
| 217753_s_at | *RPS26* | 11.87 | 11.04 | 0.83 | 0.84 | 0.56 | -0.056 |
| 211645_x_at | Unnamed transcript | 8.47 | 7.64 | 0.81 | 0.81 | 0.56 | -0.552 |
| 215176_x_at | Unnamed transcript | 9.69 | 8.86 | 0.89 | 0.84 | 0.56 | -0.498 |
| 217378_x_at | Unnamed transcript | 7.80 | 6.96 | 0.65 | 0.66 | 0.56 | -0.587 |
| 214916_x_at | *MGC27165 /// IGHG1* | 8.13 | 7.29 | 0.54 | 0.81 | 0.56 | -0.632 |
| 211644_x_at | *IGKC* | 8.67 | 7.82 | 0.76 | 0.56 | 0.55 | -0.579 |
| 218711_s_at | *SDPR* | 8.02 | 7.15 | 0.68 | 1.19 | 0.55 | -0.422 |
| 205033_s_at | *DEFA1* | 11.37 | 10.49 | 1.55 | 2.70 | 0.54 | -0.103 |
| 214677_x_at | *IGLC2* | 11.49 | 10.61 | 0.93 | 0.66 | 0.54 | -0.513 |
| 216491_x_at | Unnamed transcript | 7.51 | 6.62 | 1.00 | 0.49 | 0.54 | -0.385 |
| 211637_x_at | *MGC27165 /// IGH* | 7.87 | 6.97 | 0.82 | 0.65 | 0.54 | -0.457 |
| 215121_x_at | *IGLC2* | 10.99 | 10.08 | 0.83 | 0.54 | 0.53 | -0.558 |
| 209138_x_at | *IGLC2* | 11.43 | 10.52 | 0.95 | 0.58 | 0.53 | -0.508 |
| 212592_at | *IGJ* | 7.83 | 6.78 | 1.01 | 0.36 | 0.48 | -0.475 |
| 211430_s_at | *IGHG1* | 9.58 | 8.52 | 1.49 | 0.79 | 0.48 | -0.256 |
| 209301_at | *CA2* | 7.69 | 6.62 | 0.61 | 0.71 | 0.48 | -0.607 |
| 219629_at | *C22orf8* | 6.73 | 5.63 | 1.11 | 0.14 | 0.47 | -0.331 |
| 208601_s_at | *TUBB1* | 8.90 | 7.74 | 0.78 | 1.12 | 0.45 | -0.432 |
| 204439_at | *C1orf29* | 7.15 | 5.99 | 1.84 | 0.96 | 0.45 | -0.111 |
| 221211_s_at | *C21orf7* | 8.36 | 7.20 | 1.15 | 0.90 | 0.45 | -0.462 |
| 207815_at | *PF4V1* | 6.03 | 4.73 | 1.33 | 1.27 | 0.40 | -0.178 |
| 217022_s_at | *MGC27165* | 10.73 | 9.25 | 1.32 | 0.67 | 0.36 | -0.444 |

1. Data values given in log_2_
2. Ratio: Expression in High-Lonely / Expression in Low-Lonely. 1.30 = 10% FDR, 1.50 = 5% FDR
3. Correlation between gene expression and quantitative UCLA Loneliness score
