# Supplementary material for: Social regulation of gene expression in human leukocytes
Source: Genome Biol. 2007 Sep 13;8(9):R189. doi: 10.1186/gb-2007-8-9-r189 (PMC2375027; doi:10.1186/gb-2007-8-9-r189)
Supplement: Additional data file 2 — Results of confirmatory RT-PCR analyses verifying differential expression of selected transcripts. [file gb-2007-8-9-r189-S2.doc]

**ADF2. RT-PCR verification of microarray differential expression results.** Transcripts identified as differentially expressed in microarray analyses were independently assayed by quantitative real-time RT-PCR. Results show the fold-difference in transcript abundance (normalized to GAPDH) for high- vs. low-lonely individuals (mean ± standard error), with *p*-values indicating statistical significance by independent sample *t*-test. Multivariate analysis of variance assessed the differential expression of all transcripts simultaneously (*p* < .0001).
